# Combining Fast Orthogonal Search with Deep Learning to Improve Low-Cost IMU Signal Accuracy

**DOI:** 10.3390/s26082300

**Published:** 2026-04-08

**Authors:** Jialin Guan, Eslam Mounier, Umar Iqbal, Michael J. Korenberg

**Affiliations:** 1 Department of Electrical and Computer Engineering, Smith Engineering, Queen’s University, Kingston, ON K7L 3N6, Canadaeslam.abdelmoneem@queensu.ca (E.M.); korenber@queensu.ca (M.J.K.); 2 Electrical Engineering, College of Engineering, Illinois State University, Normal, IL 61790, USA

**Keywords:** inertial measurement unit (IMU), inertial navigation system (INS), Fast Orthogonal Search (FOS), Long Short-Term Memory (LSTM), GNSS-denied navigation, sensor error modeling, calibration, drift compensation, measurement uncertainty

## Abstract

Inertial measurement units (IMUs) in low-cost navigation systems suffer from significant drift and noise errors due to sensor biases, scale factor instability, and nonlinear stochastic noise. This paper proposes a hybrid error compensation approach that combines Fast Orthogonal Search (FOS), a nonlinear system identification technique, with deep Long Short-Term Memory (LSTM) neural networks to improve IMU signal accuracy in GNSS-denied navigation. The FOS algorithm efficiently models deterministic error patterns (such as bias drift and scale factor errors) using a small training dataset, while the LSTM learns the IMU’s complex time-dependent error dynamics from much longer training data. In the proposed method, FOS is first used to predict the output of a high-end IMU based on that of a low-end IMU, and the trained FOS model is then used to extend the training data for an LSTM-based predictor. We demonstrate the efficacy of this FOS–LSTM hybrid on real vehicular IMU data by training with a limited segment of high-precision reference measurements and testing on extended operation periods. The hybrid model achieves high predictive accuracy for predicting the high-end signal based on the low-end signal, with a mean squared error below 0.1% and yields more stable velocity estimates than models using FOS or LSTM alone. Although long-term position drift is not fully eliminated, the proposed method significantly reduces short-term uncertainty in the inertial solution. These results highlight a promising synergy between model-based system identification and data-driven learning for sensor error calibration in navigation systems. Key contributions include FOS-based pseudo-label bootstrapping for data-efficient LSTM training and a navigation-level evaluation illustrating how signal correction impacts dead reckoning drift.

## 1. Introduction

Autonomous navigation in challenging environments, including indoor spaces, underground facilities, and dense urban canyons where Global Navigation Satellite System (GNSS) signals are degraded or unavailable, requires reliable positioning, navigation, and timing (PNT) capabilities. Conventional GNSS-based solutions are vulnerable to signal blockage, multipath, jamming, and spoofing, motivating the development of alternative navigation approaches [1]. Inertial navigation remains the only fully self-contained modality that continuously measures motion independent of external infrastructure or environmental features [2].

Low-cost micro-electro-mechanical system (MEMS) inertial measurement units (IMUs) are widely deployed in autonomous vehicles, robotics, and mobile sensing platforms. However, their bias instability, scale factor errors, and stochastic noise accumulate through inertial integration, leading to rapid drift in attitude, velocity, and position estimates [1,3]. While high-grade IMUs mitigate these effects, their cost limits large-scale deployment, creating a fundamental gap between affordability and navigation performance.

Traditional mitigation strategies, such as Kalman filtering with stochastic error models (e.g., random walk or Gauss–Markov processes), often fail to capture the nonlinear and time-varying behavior of low-cost MEMS sensors [3,4,5]. As a result, advanced modeling approaches, including nonlinear system identification and data-driven learning techniques, have been investigated to better characterize IMU error dynamics [4].

Deep learning approaches, including recurrent neural networks (RNNs) and Long Short-Term Memory (LSTM) networks, have demonstrated strong capability in modeling nonlinear temporal error patterns [5,6]. For example, Li et al. proposed an RNN-based method for real-time MEMS gyroscope drift compensation [7], while Fang et al. developed an LSTM-based pseudo-measurement framework for navigation during GNSS outages [8]. Jiang et al. further demonstrated LSTM-based denoising of MEMS IMU signals [9]. Despite these advances, deep learning models typically require large, labeled datasets and often lack interpretability [10,11].

In contrast, Fast Orthogonal Search (FOS), introduced by Korenberg [12] and extended in [13], provides a data-efficient, interpretable nonlinear system identification approach. FOS has been successfully applied in signal modeling and system identification problems using sparse representations [4,14,15]. It can effectively capture deterministic IMU error components, such as bias and scale factor variations, with limited data [16,17]. However, FOS alone cannot fully capture stochastic and time-dependent error dynamics, particularly those arising from complex sensor noise and motion-dependent uncertainties, which have been effectively modeled using deep learning approaches for inertial odometry [2,18]. These complementary strengths motivate a hybrid modeling approach that integrates system identification with deep learning.

In this work, we propose a two-stage FOS–LSTM hybrid calibration framework for data-efficient low-cost IMU error modeling. First, FOS is trained on a short, synchronized segment of low-end/high-end IMU data to learn a compact mapping from low-cost measurements to high-grade reference outputs. Second, the trained FOS model is used to generate pseudo-high-end labels over a longer low-end sequence, expanding the training dataset for an LSTM predictor [19]. The LSTM then learns residual temporal dependencies and refines the correction, enabling deep learning-based calibration with reduced reliance on expensive reference measurements. Although related concepts such as pseudo-label learning [19] and navigation performance evaluation [20,21] have been studied, their integration into a unified, data-efficient calibration framework for low-cost IMUs has not been previously addressed. This work introduces a novel FOS–LSTM hybrid approach that bridges this gap.

The key contributions of this work are:

- Hybrid calibration architecture: a two-stage framework that integrates analytic system identification (FOS) with sequence learning (LSTM).
- Data-efficient bootstrapping via pseudo-labels: FOS is used to expand the training dataset for LSTM learning.
- Real-world validation under limited ground truth using vehicular datasets and high-grade IMU references.
- Navigation-level evaluation demonstrating that signal-level accuracy does not always translate to improved inertial navigation performance.
- Interpretability of IMU error structure through analysis of FOS-selected model terms.

In addition to inertial navigation, GNSS-denied positioning can be supported by complementary approaches such as signals of opportunity (SoOp) and low Earth orbit (LEO) satellite-based navigation [22,23]. However, these approaches depend on external signals or environmental features. In contrast, inertial navigation provides fully autonomous operation but suffers from drift accumulation when low-cost MEMS IMUs are used. Therefore, improving IMU signal accuracy remains essential for robust multi-sensor navigation systems.

The remainder of this paper is organized as follows. Section 2 reviews IMU error characteristics and related work. Section 3 presents the proposed methodology. Section 4 describes the experimental setup. Section 5 presents results and analysis. Section 6 discusses implications and limitations, and Section 7 concludes the paper.

## 2. Background and Related Work

This section reviews key background concepts relevant to the proposed framework, including the error characteristics of low-cost MEMS IMUs, the Fast Orthogonal Search (FOS) system identification method, and the use of LSTM networks to model inertial sensor errors. Low-cost MEMS IMUs exhibit both deterministic imperfections (e.g., bias offsets, scale factor errors, cross-axis coupling, and nonlinearities) and stochastic processes (e.g., thermal noise, flicker noise) that evolve over time. Because inertial navigation integrates these errors, even small biases can accumulate into large attitude, velocity, and position drift during GNSS outages. Effective compensation, therefore, needs to address both structured deterministic components and time-dependent stochastic dynamics.

Recent studies have explored hybrid learning, sensor fusion, and deep learning-based navigation enhancements across various domains [24,25,26,27,28,29,30,31,32,33], further motivating integrated modeling approaches. In particular, hybrid navigation frameworks that combine learning-based models with classical estimation techniques have demonstrated improved robustness under GNSS-degraded conditions [34]. Furthermore, model-reduction techniques for nonlinear systems [35] and parallel cascade identification strategies for integrated navigation systems [36] have demonstrated the effectiveness of structured modeling approaches in capturing complex system behavior, thereby reinforcing the motivation to combine system identification with learning-based methods.

### 2.1. Fast Orthogonal Search (FOS) for Nonlinear System Identification

Fast Orthogonal Search (FOS) is an algorithm for nonlinear system identification and time series modeling, originally developed by Korenberg in the late 1980s [12]. FOS builds a predictive model by selecting a subset of terms from a large dictionary of candidate basis functions (which can include past inputs, past outputs, and nonlinear combinations thereof). It uses an efficient orthogonalization procedure (related to a modified Gram–Schmidt or Cholesky decomposition) to evaluate candidate terms and avoid redundancy in the selection. The goal is to incrementally construct a parsimonious model that minimizes mean squared error (MSE) between the model output and the desired output.

FOS processes the IMU signals, as explained in more detail in [12,13,16]. We assume that both input x[n] and output y[n] are available for n=0,…,N. For the least-squares approach, we will express the output y[n] as:

(1) $$y[n]=\sum_{m=0}^{M}a_{m}P_{m}[n]+e[n]\;\;\;\;\;\;n=0,\ldots ,N,$$

once the model terms $P_{m}[n]$ are known. Here, $e\left[n\right]$ is the model error, and $P_{0}[n]=1$, but the latter is only for convenience and not mandatory. For *M* ≥ 1, FOS tests candidate functions and selects model terms of the form:

(2) $$P_{m}\left[n\right]=y[n-k_{1}]\ldots y\left[n-k_{i}\right]\;x[n-l_{1}]\ldots x[n-l_{j}]$$

Here

$$i\geq 0,\;1\leq k_{1}\leq K,\ldots ,\;1\leq k_{i}\leq K,$$

$$j\geq 0,\;0\leq l_{1}\leq L,\;\ldots ,\;0\leq l_{j}\leq L$$

Many other forms of candidate terms are possible, such as those involving sinusoids and chirp signals.

The basic structure of FOS in this study is

$$y\left[n\right]=F[y\left[n-1\right],\ldots ,y\left[n-K\right],x\left[n\right],\ldots ,x\left[n-L\right]]$$

For the models considered here, *F* is a multidimensional polynomial of 2nd degree (or 2nd order), *K* is the maximum delay in *y*, and *L* is the maximum delay in *x*. Our proposed approach used *L* = *K* = 10. This allowed hundreds of possible candidate choices for the model, yet FOS succeeded in finding very concise models that outperformed those found by LSTM nets.

An example of a simpler system is

$$y\left[n\right]=a_{0}+a_{1}\;y\left[n-1\right]+a_{2}\;x\left[n-1\right]+a_{3}\;x\left[n\right]x[n-2]+a_{4}y\left[n-1\right]y[n-2]$$

The Results and Analysis section shows the actual nonlinear models returned by FOS.

One advantage of FOS is that it can handle sparse nonlinear systems well; it finds a compact model if the system has only a few dominant nonlinearities. It also naturally accommodates missing data or uneven sampling intervals by using only the available data in the computations [12]. FOS has been applied across diverse domains, including biomedical signal processing, structural health monitoring, and control [4,14,15].

In summary, FOS provides a transparent, data-efficient way to identify deterministic error patterns in sensors. For a low-cost IMU, FOS can readily capture effects like a bias term or a scale factor error (which might manifest as a near-linear relationship between low-end and high-end sensor readings). By using polynomial terms, FOS can also model slight nonlinearities in the response. Importantly, FOS typically requires far fewer training samples than a neural network to fit these simple structures [13,16]. However, because FOS builds a specific parametric model, it may not capture all the nuances of the sensor’s behavior if the unmodeled dynamics are complex. This motivates coupling FOS with a more flexible model (like an LSTM) to handle what FOS leaves unexplained.

### 2.2. LSTM Networks for Inertial Sensor Error Modeling

Deep learning, especially recurrent neural networks, has emerged as a powerful tool for modeling time series data in navigation and sensor fusion [5]. The Long Short-Term Memory (LSTM) network is an RNN capable of learning long-term dependencies in sequence data through its gated cell architecture. Each LSTM cell contains an internal state and employs gating units (input, forget, and output gates) that regulate the flow of information. This design enables LSTMs to overcome the vanishing gradient problem and preserve information across long sequence lengths. LSTMs have proven effective for many sequence modeling tasks and have been applied to processing inertial sensor data in recent years.

In the context of inertial navigation and IMU error compensation, researchers have explored various LSTM-based approaches. One line of work integrates learning with state estimation filters; for example, Li et al. (2021) developed an RNN-enhanced unscented Kalman filter that learns to estimate and compensate for MEMS gyro random drift in real time [6]. Their approach augments the state vector with an RNN’s output to improve drift correction beyond the traditional noise models. Other researchers have used pure learning models to bridge GNSS outages. For example, Fang et al. designed an LSTM-based approach to generate pseudo-position updates for an INS during GNSS signal outages, enabling inertial navigation to continue when satellite signals are unavailable [7]. Deep learning techniques have also been applied to denoise MEMS IMU measurements. Jiang et al. demonstrated that an LSTM-RNN can effectively filter sensor noise from MEMS IMU outputs, improving signal quality for downstream navigation processing [8]. In human motion applications, LSTM models have been trained on inertial sensor data to estimate biomechanical parameters (e.g., inclination angles) with good accuracy [33].

These successes illustrate that LSTMs can model the complex error characteristics of IMUs that vary over time, with motion dynamics, and in the environment. Unlike a static regression or calibration curve, an LSTM can, in principle, learn how the IMU error at time *n* depends on the recent history of sensor readings (and possibly other contextual inputs). Deep networks are not limited by a predetermined model structure, which is an advantage when the true error dynamics are unknown or highly nonlinear. However, a major limitation is the requirement for substantial training data that covers the range of operating conditions. Training an LSTM to high accuracy often demands tens of thousands of samples (or more) of ground truth data. In applications where obtaining high-end IMU data or precise reference is costly or limited to short calibration runs, a standalone LSTM might underperform due to insufficient training. Additionally, deep learning models lack the interpretability of system identification methods; an LSTM’s internal weights do not directly explain what error pattern was learned. This opaqueness can be a drawback in safety-critical systems where understanding and validating the error model is important.

### 2.3. Prior Hybrid Approaches and Novelty of This Work

Given the complementary nature of model-based and learning-based methods, a few studies have hinted at hybrid strategies. For example, Abosekeen et al. (2021) proposed a multi-level INS/GNSS integration that combined a neural network with a bias prediction scheme to improve navigation under challenging GNSS conditions [34]. In another case, researchers have used FOS in parallel with machine learning algorithms to enhance sensor signals. These efforts indicate the potential of combining data-driven models with analytic identification. Nevertheless, a cohesive integration of FOS (or similar system ID methods) with LSTM for standalone IMU drift correction has not been fully explored in prior research. Our work fills this gap by unifying these approaches and targeting improvements to a low-cost INS in the absence of GNSS.

In summary, our approach distinguishes itself by using FOS to bootstrap and inform an LSTM model. The FOS provides physical insight (e.g., confirming the presence of a nearly linear scale factor error in the gyro) and generates synthetic high-quality data to train the LSTM. The LSTM then extends the model’s capacity to aspects that FOS alone cannot easily capture (e.g., potentially complex time-correlated noise or higher-order effects). This hybrid method is particularly well-suited to scenarios with limited ground truth data, a common situation in field deployments of sensor systems. The following sections detail how this is implemented and evaluated on real sensor data. These observations highlight the complementary strengths of model-based system identification and data-driven learning approaches. The methodology presented in Section 3 integrates these ideas by combining FOS and LSTM models to achieve data-efficient calibration of low-cost IMU signals.

## 3. Methodology

In this section, we describe the methodology for developing and evaluating the FOS–LSTM hybrid model for IMU error compensation. We first outline the dataset used and the preprocessing steps. Then, we present the FOS modeling procedure for the gyroscope signal, followed by the design of the LSTM network. Finally, we explain how the hybrid integration is performed, where FOS and LSTM are combined to produce the final enhanced IMU output. These sensing platforms represent common configurations in practical multi-sensor navigation systems, combining low-cost vision-integrated IMUs with high-grade reference navigation units [37,38,39].

### 3.1. Dataset and Preprocessing

We used two vehicular trajectory datasets collected in an outdoor driving scenario, referred to as Dataset 1 and Dataset 2. Each trajectory was recorded using two rigidly mounted inertial sensing systems (see Table 1): a low-cost MEMS IMU integrated in the Stereolabs ZED-2i stereo camera platform (Stereolabs Inc., San Francisco, CA, USA) [37] and a high-grade reference navigation system NovAtel PwrPak7-E1 GNSS/INS receiver (NovAtel Inc., Calgary, AB, Canada) [38] equipped with a KVH-1750 fiber-optic gyroscope (FOG) IMU (KVH Industries, Inc., Middletown, RI, USA) [39]. Both systems provide six-axis inertial measurements consisting of three-axis accelerometer outputs ($f_{x}$, $f_{y}$, $f_{z}$) in m/s^2^ and three-axis angular measurements ($\omega_{x}$, $\omega_{y\;}$, $\omega_{z}$) in deg/s. The NovAtel reference system provides high-accuracy inertial and GNSS measurements, which are used as ground truth for evaluation. The original IMU sensors operated at different native sampling rates. To simplify synchronization and ensure consistent model inputs, all inertial measurements were downsampled to a common rate of 50 Hz, which matches the reference navigation solution frequency used in the evaluation. In addition, vehicle forward speed measurements were obtained through an OBD-II interface (Elm Electronics Inc., London, ON, Canada) [40], providing odometer-based speed data sampled at 3 Hz to support vehicle motion analysis. The specifications of the sensors and measurement systems used in the experiments are summarized in Table 1. The datasets used in this study were collected as part of the NavINST dataset development effort for inertial navigation research, which provides synchronized multi-sensor data for evaluating navigation algorithms under realistic vehicle motion conditions [20].

In this work, we focus primarily on the gyroscope *z*-axis (azimuth) rotational rate, denoted $\omega_{z}$ (which corresponds to heading rate or yaw rate in the navigation context). Among the 6 IMU signals, the heading gyroscope is often the most crucial for maintaining accurate orientation and thus a correct heading over time; errors in heading integration can lead to large position errors in dead reckoning. Indeed, initial analyses confirmed that improving $\omega_{z}$ yields the most significant navigation benefit. For land vehicle navigation, the yaw-rate gyroscope $\omega_{z}$ plays a dominant role because vehicle motion is largely constrained to a horizontal plane; therefore, heading errors accumulate rapidly and strongly influence horizontal position drift. Therefore, while our methods were applied to all axes in principle, we report detailed results for the $\omega_{z}$ channel (and later provide a brief evaluation on an accelerometer axis for comparison).

Each trajectory’s data were partitioned for model training and evaluation as follows. Dataset 1 contains more than 40,000 samples recorded at 50 Hz, corresponding to approximately 24 min of vehicle motion, and was used for model development. From Dataset 1, the initial portion of the data was used to train models with varying training lengths (e.g., 5000, 6000, and 7000 samples), while the remaining segment was reserved for within-trajectory testing to evaluate interpolation and short-term extrapolation performance.

Dataset 2 contains approximately 13,000 samples and was reserved exclusively as an independent test dataset to assess cross-trajectory generalization of the proposed models. Dataset 2 was collected under similar environmental conditions to Dataset 1 but along a different vehicle route, providing an unseen trajectory with distinct motion dynamics to evaluate model robustness.

Before model development, the sensor data were carefully preprocessed. Both the low-cost MEMS IMU and the high-grade KVH-1750 reference IMU produced time-stamped measurements at 50 Hz; no obvious outliers or discontinuities were found. We did not apply additional downsampling or filtering beyond each device’s internal processing. Instead, the two data streams were aligned to a common 50 Hz timeline using hardware-synchronized timestamps, ensuring that all comparisons and model training steps were performed on synchronized records. The KVH-1750 IMU served solely as an offline reference for labeling training data and computing evaluation metrics; the proposed method does not perform real-time multi-IMU fusion.

Synchronization and sampling rate mismatch are critical considerations when fusing data from multiple IMUs or other sensors. Each IMU operates with its own clock oscillator, and even small frequency differences can accumulate into significant sample-count discrepancies over long recording sessions. Coviello et al. [41,42] reported that interrupt-driven sampling can produce misaligned data because IMU clock frequencies may vary by approximately ±2% and are subject to internal processing delays. To mitigate this issue, they proposed a master–slave synchronization architecture in which a master node periodically broadcasts its real-time clock (RTC) to slave units, ensuring that all IMU boards share a common synchronized timestamp. These findings highlight the importance of addressing sampling rate mismatches through hardware synchronization or software clock compensation mechanisms and provide context for the offline alignment procedure adopted in this study.

In our workflow, synchronization is handled offline: models are trained and evaluated using the aligned 50 Hz records. We intentionally did not apply additional calibration to the low-cost IMU beyond the manufacturer’s processing, since the objective is for the proposed models to learn the mapping from noisy low-cost measurements to high-grade reference outputs. The KVH-1750 data, characterized by substantially lower noise and drift, are, therefore, treated as the ground truth reference signal. This offline synchronization ensures that comparisons between low-end and reference IMU signals are free from timing artifacts that often affect real-time multi-IMU fusion systems.

For the LSTM training stage described later, Dataset 1 was further divided into training and validation subsets as required. All input features were standardized using statistics computed from the training set, and the same normalization was applied to test data to ensure consistent scaling and stable network training. The LSTM receives only inertial measurements and learns the error mapping between the low-cost IMU outputs and the high-grade reference signals.

### 3.2. FOS Modeling of Gyroscope Error

First, we employed Fast Orthogonal Search to model the relationship between the low-end and high-end readings on the gyroscope *z*-axis. The FOS algorithm was given the low-end $\omega_{z}$ time series as input *x*[*n*] and the high-end $\omega_{z}$ as the desired output *y*[*n*]. We configured FOS to consider constant and polynomial terms up to second order in the current input (and optionally a few past samples) as candidate basis functions. In practice, we found that including past samples (lag) did not significantly improve the fit for this specific signal, so the FOS model effectively utilized only the current sample $x\left[n-0\right]$ in the chosen terms. FOS was configured to stop adding terms if the improvement in mean squared error (MSE) fell below a threshold (on the order of $10^{-4}$) or if a maximum of 5 terms were selected, to prevent overfitting.

**Figure 1:**
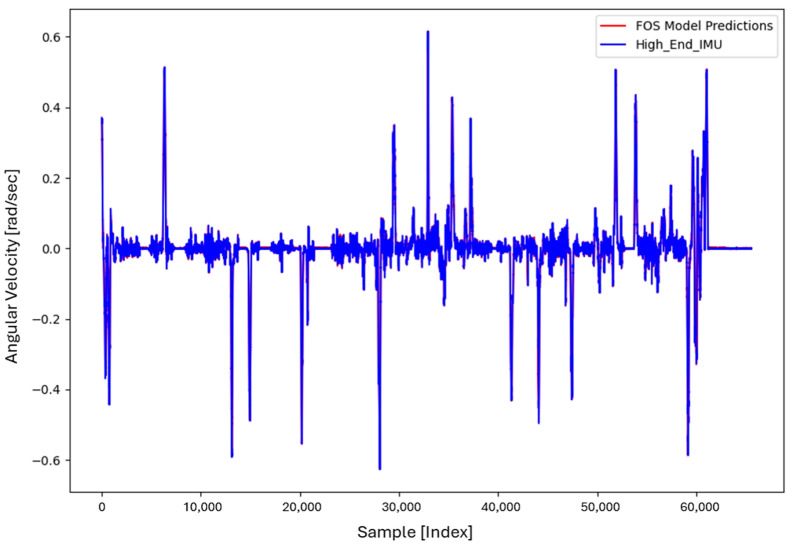
FOS prediction of high-end $\omega_{z}$ on a novel portion of Dataset 1 after training FOS on the first 6000 samples of Dataset 1. The FOS output (orange) closely matches the ground truth high-end gyro (blue). The prediction error is too small to see at this scale (%MSE ≈ 0.0146%).

**Figure 2:**
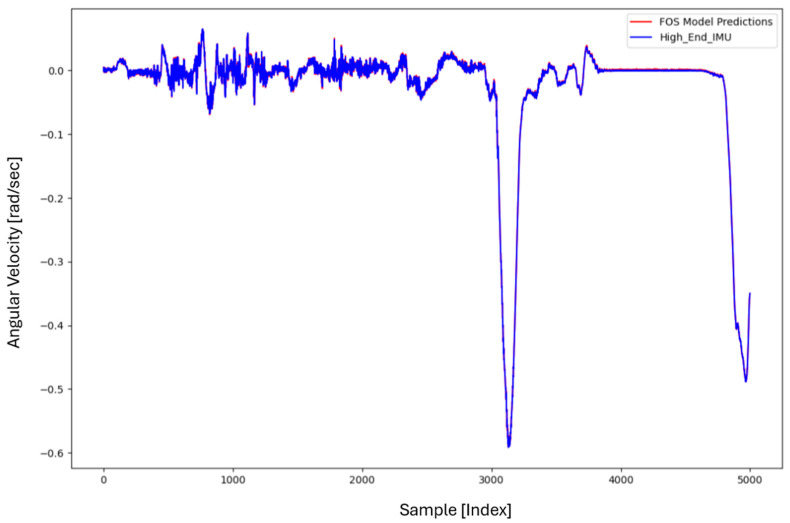
Zoomed-in view of Figure 1, highlighting a segment of the $\omega_{z}$ time series. Even in the zoomed view, the low error of the FOS prediction is evident (the two curves overlap almost completely).

**Figure 3:**
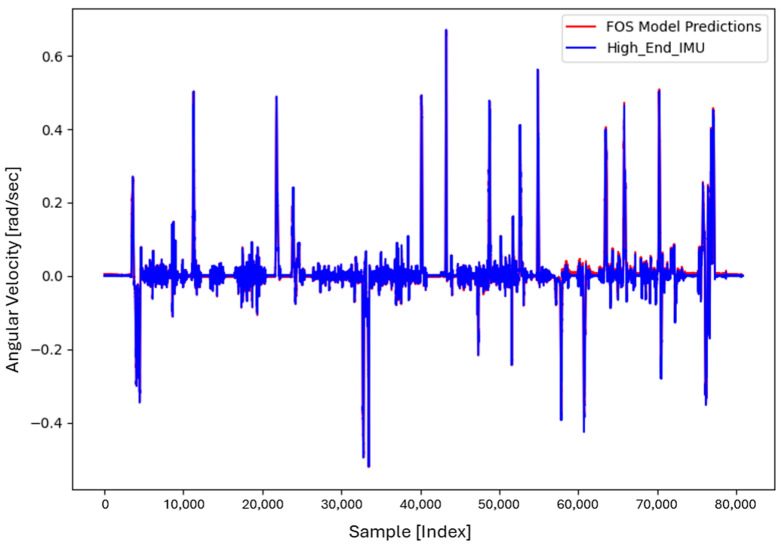
FOS predicted high-end $\omega_{z}$ for the entirety of Dataset 2 (unseen trajectory) using the model trained on 6000 points from Dataset 1. The prediction (orange) remains very accurate relative to the actual high-end signal (blue), with %MSE ≈ 0.0150% on Dataset 2.

**Figure 4:**
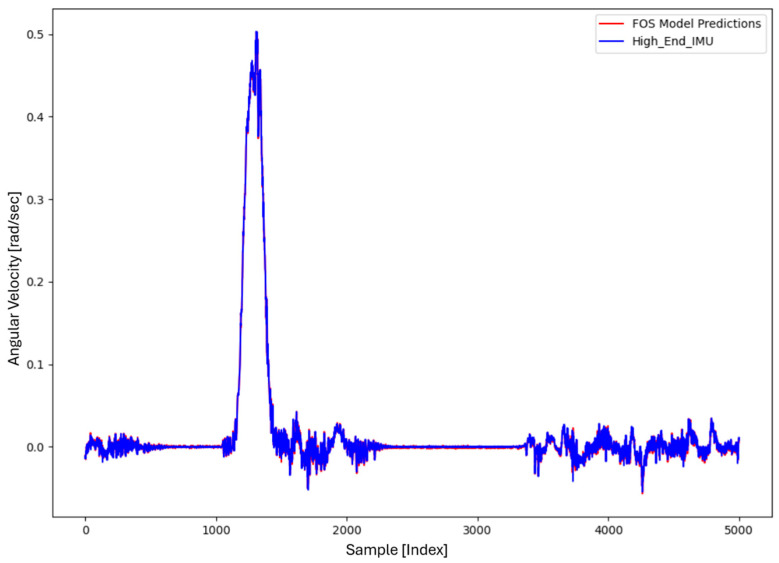
Zoomed-in view of the FOS prediction vs. actual $\omega_{z}$ on Dataset 2. The FOS model maintains high accuracy, indicating it captured the primary calibration difference between the low-cost and high-end gyro.

**Figure 5:**
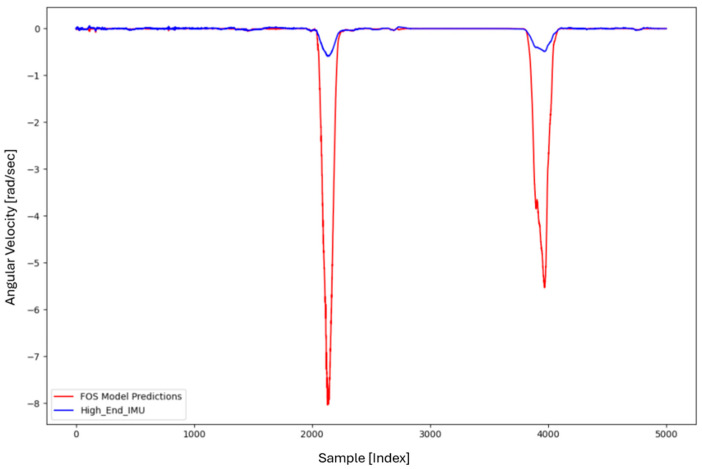
Zoomed view of the FOS prediction of high-end $\omega_{z}$ using 5000 training samples from Dataset 1, illustrating significant prediction error compared with the reference signal.

**Figure 6:**
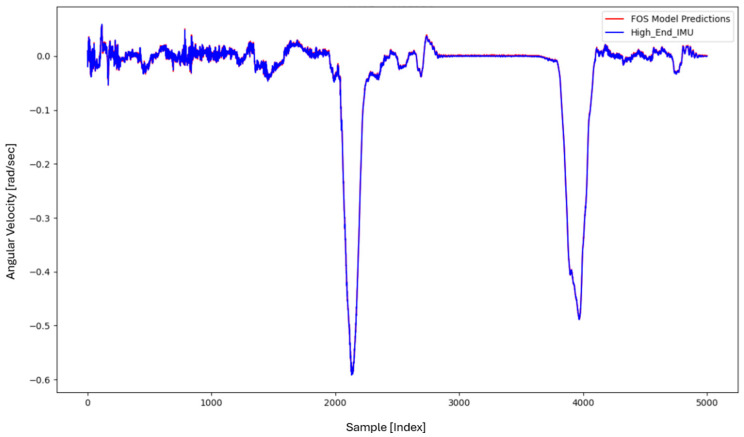
Zoomed view of the FOS-predicted $\omega_{z}$ on unseen samples of Dataset 1 using 7000 training samples, showing improved agreement with the high-end reference signal.

For the core training of FOS, we experimented with using N=5000 and N=7000 sample pairs from the start of Dataset 1. Table 2 summarizes the FOS models obtained. Figure 1, Figure 2, Figure 3 and Figure 4 illustrate FOS predictions for the yaw-rate channel on Datasets 1 and 2 (Figure 1 shows the full prediction on Dataset 1, Figure 2 provides a zoomed view, and Figure 3 and Figure 4 present corresponding results for Dataset 2), demonstrating the accuracy of the FOS model. With N=5000, FOS returned a quadratic model (Model 1) containing a large negative coefficient −22.7 that yielded a poor fit on new data (%MSE ≈ 76.99%, see Figure 5). In contrast, with N=7000, FOS identified an almost perfectly linear relationship (Model 3) defined as $y[n]\approx 0.0003+0.9991\;x[n]-0.0008\;{x[n]}^{2}$, achieving an extremely low error on held-out data from Dataset 1 (%MSE ≈ 0.0072%, Figure 6).

However, we elected to use an intermediate training length of 6000 points for subsequent hybrid experiments. The comparison in Table 2 illustrates the reason at 6000 points, FOS Model 2 was essentially y[n]=0.0003+0.9997x[n], a near-identity mapping with a slight bias, and reached a %MSE of ≈0.01458% on Dataset 1 and 0.01499% on Dataset 2. Meanwhile, as discussed in Section 6, an LSTM trained on 6000 points performed much worse ( MSE ≈ 48.72%), whereas at 7000 points, the LSTM dramatically improved to ≈3.22% error. Thus, using 6000 points provides a scenario where FOS is highly accurate, but a same-sized LSTM is not, presenting a challenging case to demonstrate the benefit of the hybrid approach.

FOS Model 2 (6000-point training) served as the basis for generating additional data for the LSTM. Figure 1 shows the FOS prediction on a novel portion of Dataset 1 (unseen during training) using Model 2, and Figure 2 is a zoomed view; the FOS-corrected signal overlaps almost exactly with the high-end ground truth. Figure 3 and Figure 4 show FOS Model 2 applied to Dataset 2, indicating a similarly good fit (slightly higher %MSE but still on the order of $10^{-2}\%$). These plots illustrate the very low prediction errors achieved by FOS for $\omega_{z}$ with only 6000 training samples.

From these FOS results, we learn that the low-end gyro’s $\omega_{z}$ error relative to the high-end is largely captured by a simple linear model. With enough training data (around 6000 points in this case), FOS essentially finds y≈x, meaning the low-end and high-end gyros were almost identical in scale. This might imply that the low-end gyro was reasonably calibrated for scale but perhaps had a tiny bias. The 5k sample model was insufficient, likely because with less data, FOS latched onto some spurious nonlinearity (the $-22.7\;x^{2}$ term) that does not generalize. The jump from 76% error to 0.014% error between 5000 and 6000 samples is striking. It suggests that a nonlinearity might have been falsely included when data were limited, but more data allowed FOS to discard that and settle on the true linear relationship.

For completeness, we also examined FOS models for the accelerometer signals (denoted $f_{x},\;f_{y},\;f_{z}$ for specific forces along the body axes). The low-end accelerometers had more noise and possibly other issues, so FOS models did not achieve as low an error as with the gyro. For example, using 7000 training samples for the $f_{x}$ (*X*-axis accelerometer), FOS returned a model y[n] = 0.1373 + 0.9390 x[n] + 0.0023 ${x[n]}^{2}$, which had about 24.55% MSE on the new points of Dataset 1. This indicates that the accelerometer had a bias (≈0.1373) and a scale factor (≈0.939) error, plus a small nonlinearity. Still, a 24% error is fairly high, meaning other unmodeled factors (perhaps noise or more complex dynamics) remained. We found that trying to use such a model directly for a hybrid approach would not be as fruitful unless the FOS stage can achieve a lower error. In this paper, we primarily focus on the gyro $\omega_{z}$ case, where FOS performs excellently, and briefly report the results on $f_{x}$ to illustrate the method’s applicability and limitations beyond the best-case scenario.

### 3.3. LSTM Network Design and Training

Next, we designed a Long Short-Term Memory (LSTM) neural network to learn the mapping from low-end IMU data to high-end IMU data. The LSTM is used in two contexts: (a) as a standalone model trained purely on available high-end data, to compare against FOS, and (b) as part of the hybrid approach, where it is trained on an augmented dataset with FOS-generated outputs.

Network Architecture: The predictor is implemented using the TensorFlow/Keras deep learning framework with a compact sequence model designed for efficient training and low-latency inference. The input layer is a sequence layer that processes the time series IMU measurements. This is followed by a primary LSTM layer with 64 hidden units (approximately 16,896 parameters) to capture temporal dependencies in the inertial sensor data. The LSTM layer is followed by a fully connected dense layer with 8 hidden units using the Rectified Linear Unit (ReLU) activation function, defined as

ReLU(x)=max(0,x)

The final layer is a dense output layer with a linear activation function producing a scalar prediction corresponding to the corrected gyroscope signal. The input to the network at each time step consists of the low-end IMU measurement (specifically $\omega_{z}$) synchronized with the corresponding high-end reference signal.

Hyperparameters: The network was trained using the mean squared error (MSE) loss function. The learning rate was set to 0.0001, and the root mean squared error (RMSE) was used as the validation metric for model selection. The total number of training epochs was set to 100, and the parameters were tuned empirically to achieve stable convergence and reliable prediction performance.

Architecture and Hyperparameter Rationale: The selected configuration reflects an empirical trade-off between modeling capability and computational efficiency. Several representative alternatives were evaluated during preliminary experiments, including LSTM layers with 32, 64, and 128 hidden units, as well as single-layer and two-layer recurrent architectures. Learning rates in the range of $10^{-3}$ to $10^{-4}$ were also explored. The final configuration was selected based on validation performance measured using %MSE and RMSE on held-out segments of Dataset 1. The chosen architecture corresponds to the smallest model that achieved near-optimal validation accuracy, reducing the risk of overfitting while maintaining sufficient capacity to capture the temporal dynamics of IMU error behavior. These experiments showed that increasing the number of hidden units beyond 64 produced only marginal improvements in prediction accuracy while increasing computational cost and risk of overfitting; therefore, the 64-unit configuration was selected as the most balanced architecture for this study. This choice aligns with the objective of this work, which emphasizes data-efficient calibration of low-cost IMU signals rather than maximizing neural network complexity.

Standalone LSTM Performance on Varying Training Sizes: To provide a baseline and understand data requirements, we trained standalone LSTM models on different amounts of high-end training data from Dataset 1: 5000, 6000, 7000, 10,000, and 40,000 points. In each case, the training data started from the beginning of Dataset 1. The largest model (40,000 points) utilized a validation set of 5000 points and was tested on the remaining unseen portion of Dataset 1, as well as the entirety of Dataset 2, to assess generalization.

Observations: Several important observations can be made from Table 3. First, the LSTM requires a large amount of data to reach high accuracy. With only 5000 points, LSTM Model 1 performs very poorly (%MSE ~ 83.6%, essentially unusable). Even at 6000 samples (Model 2), it is underfitting significantly (48.72% error, $R^{2}\approx$ 0.51). We see a dramatic improvement by 7000 points; the error drops to ≈3.2% and $R^{2}$ jumps to 0.968. This suggests a threshold around 7000 samples where the network starts to effectively capture the relationship. Increasing to 10,000 points further pushes the error below 0.4%. With an extensive 40,000 sample training set, the LSTM achieves an almost perfect fit on the test data (0.017% error, $R^{2}$ ≈ 0.999).

Table 3 summarizes the results of these LSTM models and the final hybrid model. We list the training length, the achieved percent MSE (%MSE), and the coefficient of determination ($R^{2}$ score) on the respective test sets.

These results underscore the data-intensive nature of the deep model; it took on the order of tens of thousands of samples for the LSTM to match the tiny error that FOS achieved with only 6000 samples (recall FOS with 6000 samples had ≈0.0146% error). Indeed, the 40,000 sample LSTM’s 0.0174% error is in the same ballpark as the FOS model’s 0.0146%. The hybrid model, however, achieved the lowest error of all (0.01393%) by leveraging the strengths of both approaches.

When applied to Dataset 2, the 40,000-point LSTM achieved an $R^{2}$ of 0.99855 and a %MSE of 0.144%. While this represents a slight degradation compared to the test on Dataset 1 (0.017%), 0.144% remains a very low error in absolute terms, demonstrating the model’s robustness.

### 3.4. FOS–LSTM Hybrid Integration

The core idea of our hybrid approach is to leverage FOS to either assist or enhance the LSTM training. There are two possible modes of integration we considered:

- Sequential Error Correction: In this mode, the FOS model and LSTM model are applied in series. The low-end sensor reading is first passed through the FOS model (which corrects deterministic bias/scale errors), and then the LSTM takes the FOS-corrected signal as input (optionally with the original) to predict the remaining error. This way, the LSTM only needs to learn the residual stochastic errors after the bulk systematic errors have been removed by FOS. We implemented this concept by training an LSTM on the difference between the high-end signal and the FOS prediction. However, in our experiments, we found that if FOS already achieved extremely low error (as in $\omega_{z}$ case with 6 k training), the residual errors were almost noise-level, and the LSTM had difficulty learning much (risk of overfitting noise). This approach might be more useful if FOS leaves more significant residuals (e.g., in the accelerometer case, where FOS had 20–30% error, an LSTM could target that residual). In the gyro $\omega_{z}$ case, sequential correction did not yield noticeable improvement over FOS alone (because there was not much left to correct on Dataset 1; on Dataset 2, the residual might be more, but we opted for the second integration mode for better generalization).
- Data Augmentation (Pseudo-Ground Truth Generation): In this mode, the FOS model is used to expand the training dataset for the LSTM. Specifically, after training FOS on a small set of high-end data (say, the first *N* samples of Dataset 1), we use the low-end IMU data beyond that (from sample *N* + 1 onward, potentially through the rest of Dataset 1 or even other datasets) as input to the FOS model to predict the high-end output. These FOS-predicted outputs serve as a surrogate for true high-end measurements. By combining the original *N* true pairs with these additional synthetic pairs, we assemble a much larger training set for the LSTM. The LSTM is then trained on this combined dataset, with the hope that it learns the mapping with the help of quantity (the additional data), even if some of that data are approximate. Essentially, FOS is used to generate labels where none were available, bootstrapping the deep learning. This is the approach we found most effective for the $\omega_{z}$ case, which we highlight in our results as the “Hybrid model.”

For the hybrid results reported, we chose *N* = 6000 as the amount of real high-end data used. This choice was motivated by the earlier observation that at 6000, FOS was very accurate, while an LSTM trained only on 6000 was very poor (almost 50% error). This scenario presents the largest potential gain from the hybrid: we have limited real data, FOS can make good use of it, and LSTM alone cannot. For the hybrid results reported in this study, we used N=6000 samples of paired low-end and high-end IMU measurements from Dataset 1 to train the FOS model (corresponding to FOS Model 2 in Table 2). The trained FOS model was then applied to the subsequent low-end IMU samples from Dataset 1 (beyond the first 6000 points) to generate additional pseudo-high-end outputs. These FOS-generated outputs served as surrogate reference measurements for expanding the training dataset. By combining the original 6000 real paired samples with the additional FOS-generated pairs from Dataset 1, we constructed an extended training dataset of approximately 40,000 samples. An LSTM network with the architecture described in Section 3.3 was then trained on this expanded dataset. Dataset 2 was reserved exclusively for independent testing and was not used during pseudo-label generation or LSTM training, ensuring a proper out-of-sample evaluation of the hybrid model.

During training of this hybrid LSTM, the loss function does not distinguish between true and synthetic targets; it treats all 40,000 targets as ground truth. We did, however, monitor the validation error using a validation set that included a mix of real and FOS-augmented data (to ensure the network was not overfitting peculiarities of FOS outputs). The training proceeded similarly to the pure LSTM case, and we obtained a model which we refer to as the hybrid FOS–LSTM model.

At inference time, the hybrid model operates simply as an LSTM model; it takes a sequence of recent low-end $\omega_{z}$ readings and outputs a corrected $\omega_{z}$. The FOS’s role was only in creating the training data. (In an alternative usage, one could also run the FOS in parallel and average or cross-check, but we did not do that; we trust the LSTM to subsume the FOS knowledge since it was trained on FOS outputs.)

It is worth noting that this hybrid approach effectively assumes that the FOS predictions are sufficiently accurate; if FOS were to produce highly biased or wrong outputs for some region, the LSTM would then learn from flawed data. In our case, since FOS was very accurate for $\omega_{z}$, this was not a concern; the synthetic data were almost as good as real. For other signals, care must be taken (as we discuss with the accelerometer example in the results, where the hybrid did not help as much because FOS’s error was still significant).

Finally, in principle, one could combine both integration modes: use FOS correction sequentially and also train LSTM on extended data. We did not explicitly do this because if one is applying FOS first in real-time, one might just stop there unless the LSTM provides further benefit. Our experiments indicated the sequential application for $\omega_{z}$ was not beneficial since FOS already did so well. Thus, the main hybrid results are from the data augmentation strategy.

## 4. Experimental Setup

This section details the experimental setup used to evaluate the FOS, LSTM, and hybrid models. It includes the computing environment, the evaluation metrics, and the procedure for testing the impact of these models on an INS navigation solution.

Computing Environment: Model development and evaluation were performed on a computer with an Computing Environment: Model development and evaluation were performed on a computer with an AMD Ryzen 7 3700X CPU (Advanced Micro Devices, Inc., Santa Clara, CA, USA) and an NVIDIA RTX 2070 GPU (NVIDIA Corporation, Santa Clara, CA, USA). All algorithms were implemented in Python 3.8. We used the Pandas library for data handling and preprocessing, the NumPy library (version 1.23.5) for numeric computations, and Matplotlib (version 3.7.2, Matplotlib Development Team, open-source) for plotting results. The FOS algorithm was custom-implemented based on the description in Korenberg’s work; it was verified on known polynomial test cases. The LSTM network was implemented using TensorFlow/Keras. Training of the largest LSTM (40,000 points) took on the order of a few minutes on the GPU, which is relatively modest; this reflects the small network size and data size by deep learning standards. FOS training was nearly instantaneous (fractions of a second for each model), given the small number of terms and efficient implementation. During inference, the computational cost of both models is very low. The FOS model consists of a compact analytic polynomial requiring only a few arithmetic operations per sample, while the trained LSTM network contains a lightweight architecture with a single 64-unit recurrent layer. At the dataset sampling rate of 50 Hz, both models generate predictions substantially faster than real time, indicating that the proposed method is suitable for real-time IMU signal correction in practical navigation systems.

Evaluation Metrics: We primarily use two quantitative metrics to evaluate model performance: (1) mean squared error (MSE), expressed as a percentage of the variance of the desired signal (%MSE), and (2) coefficient of determination ($R^{2}$). The %MSE is the MSE divided by the variance of ground truth data multiplied by 100. The value of $R^{2}$ lies between 0% and 100%. If $R^{2}$ is 100%, then the model perfectly fits the data, and there is no difference between the predicted value and the actual value. The %MSE function is straightforward to compute from NumPy. The $R^{2}$ score function is from the Scikit-Learn metrics library.

In addition to inertial measurements, odometer-derived vehicle speed measurements were used to constrain the velocity mechanization during the dead reckoning experiments. Incorporating odometer measurements helps reduce velocity drift in ground vehicle navigation by providing an independent estimate of forward speed. This approach follows established INS/odometer integration strategies used in land vehicle navigation systems [21]. Specifically, we focused on horizontal position error during a segment where GNSS was absent (simulated). We initialized an INS at a known location and propagated it using: (a) low-end IMU data (baseline), (b) low-end IMU data, but with the low-end $\omega_{z}$ replaced by the high-end $\omega_{z}$ (best-case reference), (c) low-end IMU with $\omega_{z}$ replaced by FOS-predicted high-end $\omega_{z}$, (d) low-end IMU with $\omega_{z}$ replaced by LSTM-predicted (40,000 model) $\omega_{z}$, and (e) a low-end IMU with $\omega_{z}$ from the hybrid model. In all cases, we used the low-end accelerometers as is (no correction) to isolate the effect of gyro heading error on horizontal position. The vehicle’s motion in these datasets was largely planar (no significant elevation change), and we assumed flat-earth mechanization for simplicity. The horizontal position error RMS (root mean square) over time was computed for each case. This provides an indicator of how improvements in sensor signal accuracy translate to improved dead reckoning navigation.

Test Procedure: We trained the various models on Dataset 1 segments as described and then evaluated them on the latter part of Dataset 1 (to verify interpolation/extrapolation on the same trajectory) and on Dataset 2 (to test generalization). We plot representative portions of predictions vs. ground truth for visualization (as in Figure 1, Figure 2, Figure 3 and Figure 4 for FOS, analogous figures for LSTM, etc.). For the LSTM and hybrid models, we also compute their error metrics on Dataset 2, as this is a critical measure of performance in a GNSS-denied transfer scenario (train in one route, apply in another). Finally, we simulate a GNSS outage scenario on Dataset 2 and compute the INS horizontal error accumulation for each method.

By comparing these results, we can determine whether the hybrid approach indeed provides a benefit over using FOS or LSTM alone and quantify that benefit in terms of both sensor signal accuracy and resulting navigation accuracy.

## 5. Results

### 5.1. Sensor Signal Prediction Accuracy

FOS vs. LSTM on Gyro $\omega_{z}$: As seen earlier in Table 2 and Table 3, FOS with 6000 training points (FOS Model 2) achieved a remarkably low error on both the tail of Dataset 1 and on Dataset 2 (%MSE ≈ 0.015%). By contrast, an LSTM with 6000 training points had ≈48.7% error on the tail of Dataset 1, several orders of magnitude worse. Even with 7000 points, LSTM’s error (≈3.2%) was much higher than FOS’s, though at 7000, the LSTM was starting to get the trend right ($R^{2}$ ≈0.968). With a very large training set (40,000), the LSTM matched FOS on Dataset 1 (0.017% vs. 0.014% error). On Dataset 2, the 40,000 LSTM had 0.144% error, whereas the 6000-point FOS had 0.015%; interestingly, the FOS model generalized better to the new run than the LSTM did, even though the LSTM was trained with far more data (including some from Dataset 2). This suggests that the simple linear model captured by FOS remained consistent across runs, whereas the LSTM may have learned slightly more complex features that did not transfer as perfectly.

The stark difference at low training sizes highlights the central premise of our work: FOS can attain high accuracy with limited data, whereas deep learning initially struggles. This gap is what the hybrid method aims to bridge.

**Figure 7:**
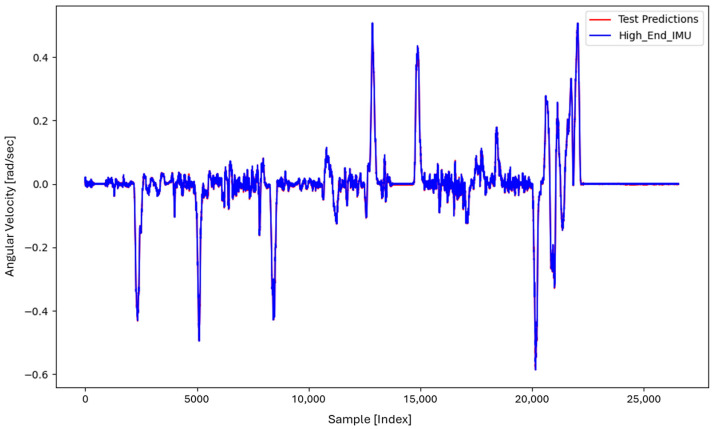
LSTM prediction of $\omega_{z}$ on unseen samples of Dataset 1 trained with 40,000 samples, demonstrating near-perfect agreement with the reference IMU signal.

**Figure 8:**
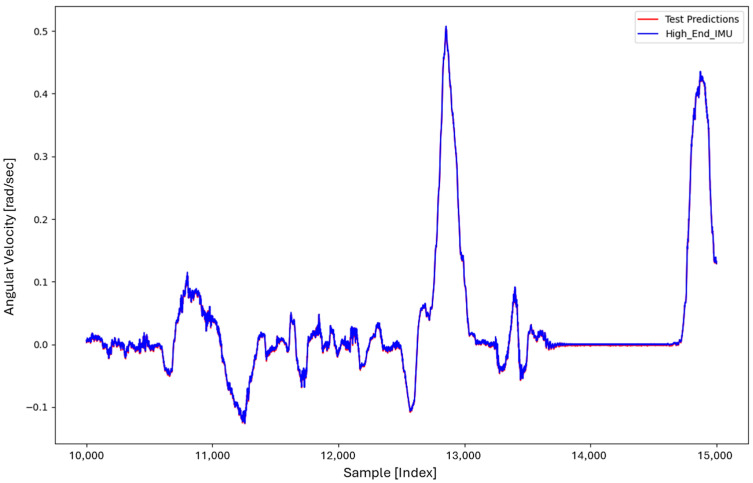
Zoomed view of the LSTM prediction of $\omega_{z}$ on Dataset 1, highlighting the very small residual error after training with 40,000 samples.

Hybrid Model Performance (Gyro $\omega_{z}$): The hybrid model was trained with effectively 6000 real + 34,000 FOS-simulated points (total 40,000). Its performance on Dataset 1’s unseen portion was excellent (%MSE 0.01393%, $R^{2}$ 0.99986, per Table 3). Essentially, on data from the same distribution it was trained on (which includes FOS outputs), it fit almost perfectly, slightly even better than the pure LSTM 40,000 model. More importantly, on Dataset 2 (completely never-before-seen by the hybrid during training), the hybrid achieved %MSE = 0.2173% and $R^{2}$ = 0.9978. This is still a very high accuracy, though it is about 1.5× the error of the pure 40,000-point LSTM on Dataset 2 (0.144%). It is also an order of magnitude higher error than the FOS model’s 0.015% on Dataset 2. In other words, in this particular case, the hybrid did not generalize as well as the simpler FOS model to the second run. It slightly underperformed the best-case LSTM as well, on that Dataset 2 test. However, it was vastly superior to any model that could have been trained solely on 6000 real points (the LSTM with 6000 real points was 48% error). Figure 5, Figure 6, Figure 7 and Figure 8 illustrate some of these comparisons.

Figure 5 shows the prediction of $\omega_{z}$ obtained using LSTM Model 1, trained with 5000 samples from Dataset 1. The predicted signal deviates substantially from the high-end IMU reference, indicating insufficient training data to capture the gyroscope error dynamics.

Figure 6 presents the prediction results of LSTM Model 3, trained with 7000 samples. The predicted signal better follows the reference trend; however, noticeable discrepancies remain, suggesting incomplete modeling of the sensor error.

Figure 7 illustrates the prediction obtained using an LSTM model trained with 40,000 samples from Dataset 1. At this training scale, the predicted signal nearly overlaps with the reference signal, indicating that the LSTM effectively captures the underlying error characteristics.

Figure 8 provides a zoomed view of Figure 7, revealing small residual differences that are not visible at the original plotting scale.

**Figure 9:**
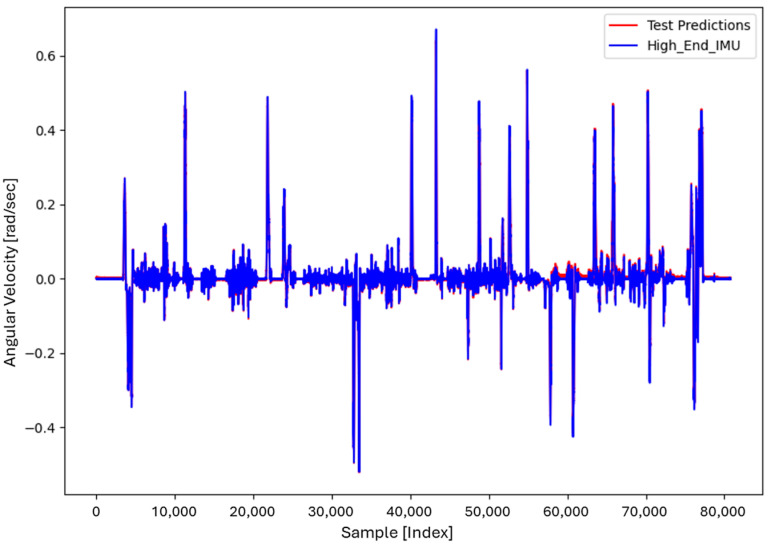
LSTM prediction of $\omega_{z}$ on Dataset 2 (unseen trajectory) using a model trained with 40,000 samples from Dataset 1, demonstrating strong cross-trajectory generalization.

To evaluate generalization, the LSTM model trained on Dataset 1 (40,000 samples) was applied to Dataset 2, as shown in Figure 9. The predicted $\omega_{z}$ closely follows the reference signal, demonstrating strong cross-trajectory generalization.

**Figure 10:**
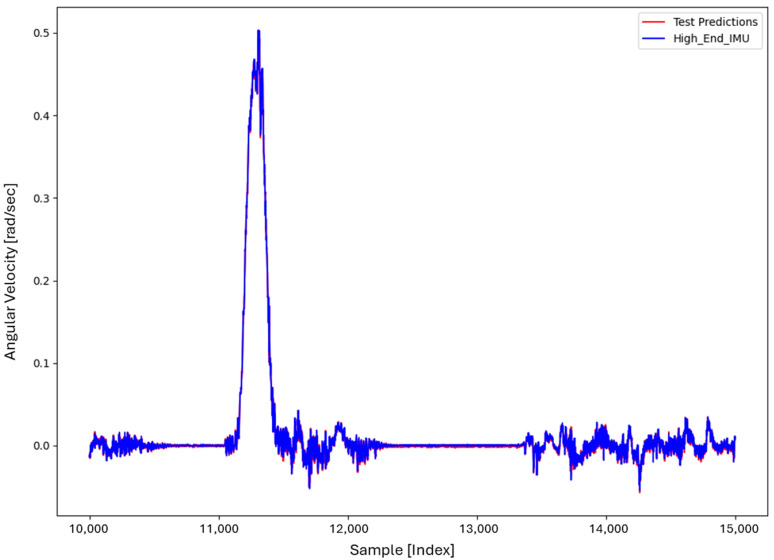
Zoomed view of the LSTM prediction on Dataset 2, illustrating the small residual prediction error (≈0.144% MSE).

Figure 10 shows a zoomed view of the Dataset 2 prediction, where minor deviations between the predicted and reference signals can be observed. The corresponding prediction error remains low, with a mean squared error of approximately 0.144%.

**Figure 11:**
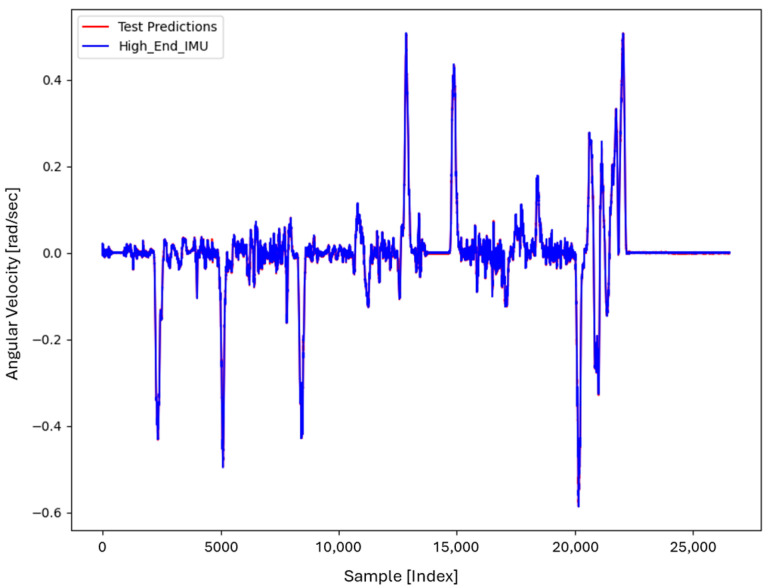
Hybrid model predicted $\omega_{z}$ on novel values of Dataset 1. The hybrid model was trained using 6000 real samples from Dataset 1 and 34,000 FOS-generated pseudo-samples, yielding an extended training set of approximately 40,000 samples.

**Figure 12:**
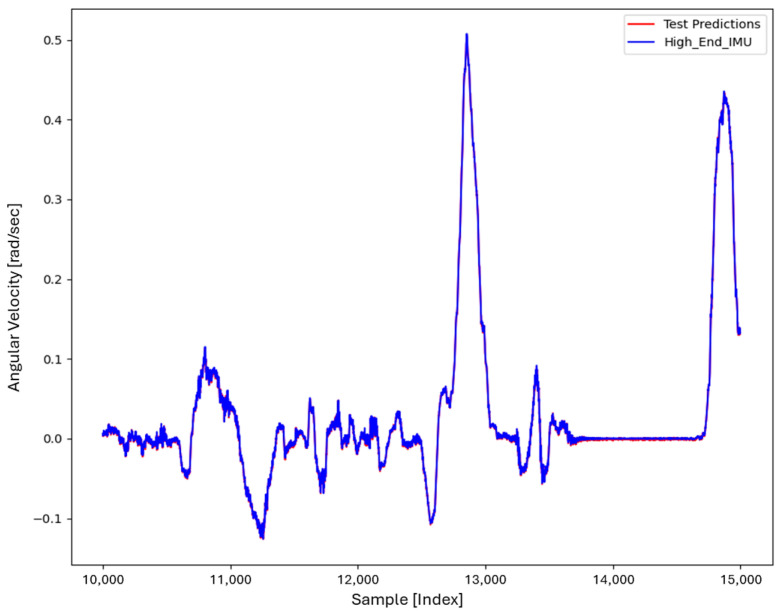
Zoomed view of the hybrid FOS–LSTM prediction of $\omega_{z}$ on Dataset 1, highlighting the small residual differences between the predicted and reference signals. The hybrid model was trained on a dataset extended from the 6000th timestep using 34,000 FOS-predicted values.

Figure 11 shows the hybrid model prediction of $\omega_{z}$ on the unseen portion of Dataset 1. The predicted signal closely overlaps with the high-end reference IMU measurement, resulting in a very small prediction error of approximately 0.0139% MSE. Figure 12 presents a zoomed view of the same segment, highlighting the small residual differences that are not visible at the original scale.

**Figure 13:**
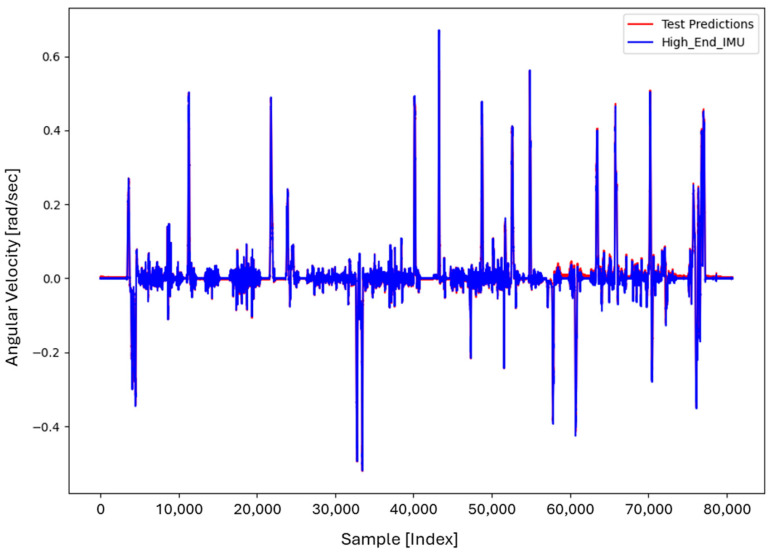
The hybrid model predicted $\omega_{z}$ over Dataset 2. The hybrid model was trained on 6000 values from Dataset 1 extended from the 6000th timestep using 34,000 FOS-predicted values.

**Figure 14:**
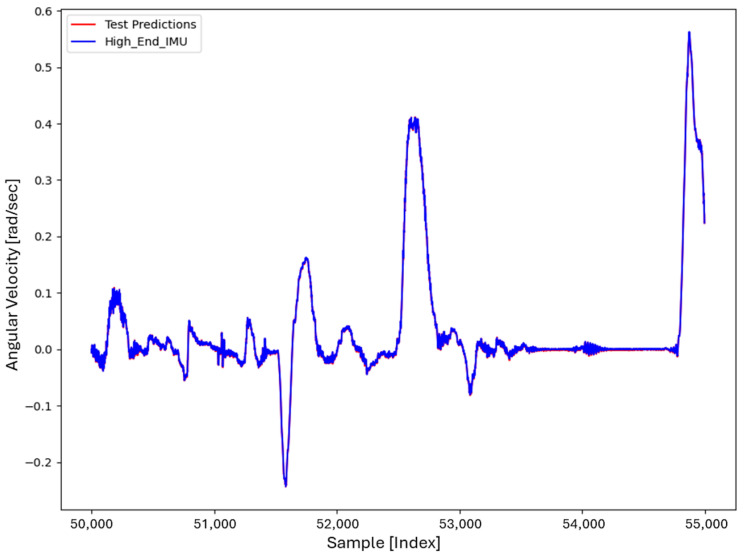
Zoomed-in plot of hybrid model prediction $\omega_{z}$ on Dataset 2, illustrating small residual deviations corresponding to a prediction error of approximately 0.217% MSE.

To evaluate cross-trajectory generalization, the hybrid model was applied to Dataset 2, as shown in Figure 13. The predicted signal remains highly consistent with the high-end reference IMU measurements. Figure 14 provides a zoomed view of this result, where small deviations between the predicted and reference signals become visible. These residual differences correspond to a prediction error of approximately 0.217% MSE, indicating that the hybrid model maintains strong prediction accuracy even on an unseen trajectory.

From these results, we conclude that the hybrid model successfully leveraged limited real data to achieve accuracy on par with a fully trained deep model. The slight generalization shortfall on Dataset 2 for the hybrid might be due to the hybrid model leaning on FOS’s structure, which might not capture a subtle difference in sensor behavior between the two runs (perhaps temperature or other factor changed between 10:31 and 11:02 a.m. runs, introducing a slight bias change that FOS’s constant model could not capture, and the LSTM trained heavily on FOS outputs would inherit that). In contrast, an LSTM trained on actual data from both runs (as our 40,000 scenario effectively did) might have been able to learn that subtlety. Nonetheless, the hybrid’s error of 0.217% is extremely low in an absolute sense.

Results on Accelerometer $f_{x}$: To illustrate the method on another sensor axis, we applied the hybrid approach to the low-end accelerometer $f_{x}$ (forward accelerometer). Here, FOS with 7000 points yielded ≈24.55% MSE on test, as mentioned. We then used that FOS model to generate 33 k more points and trained an LSTM (7 k real + 33 k FOS). On Dataset 1’s novel part, the hybrid got ≈21.56% MSE, and on Dataset 2, it got ≈23.27% MSE. These are comparable to (actually slightly higher than) an LSTM that was trained on 40,000 real high-end $f_{x}$ (which achieved ≈20.99% on Dataset 1). So, for $f_{x}$, the hybrid essentially managed to reach the performance of a fully trained LSTM with only 7 k real points. That is still a win in terms of data efficiency. However, the error levels (≈20–25%) are relatively high, indicating that $f_{x}$ errors are harder to model, likely because accelerometer errors involve not just simple bias/scale but also axis misalignments or higher noise that FOS’s second-order model could not fully capture. In such a case, the hybrid’s benefit is limited by the accuracy of FOS. If FOS’s basis functions are insufficient to explain more than ≈75% of the variance, the synthetic data will carry that 25% error, and the LSTM trained on it cannot magically do better than the data it was given. One would need either a more complex FOS model (higher-order terms or additional inputs) or some real data to cover that gap. Thus, for $f_{x}$, while the hybrid did help achieve ≈23% error using only 7000 real points (versus needing 40,000 real points to hit ≈21%), the end accuracy might not be deemed “good enough” for high-precision work. For $\omega_{z}$, in contrast, the hybrid brought us into the sub-0.3% error range, which is excellent. While the previous results evaluate prediction accuracy at the sensor signal level, the ultimate objective of IMU calibration is to improve navigation performance. Therefore, Section 5.2 evaluates how these corrected sensor signals affect the inertial navigation solution under GNSS-denied conditions.

### 5.2. Impact on Navigation Solution

The ultimate goal is to improve IMU signals to enhance navigation performance when GNSS is unavailable. To evaluate this, we substituted the low-end gyroscope’s yaw rate $\omega_{z}$ with the outputs from our different models and ran a dead reckoning INS over a GNSS outage segment of Dataset 2. We focused on horizontal error growth, since heading drift dominates in flat terrain scenarios.

Using the raw low-end MEMS IMU, the horizontal position error increased rapidly due to uncompensated gyroscope bias and heading drift. After approximately four minutes of dead reckoning, the horizontal RMS error reached 399.244 m, while the vertical RMS error was 87.307 m, illustrating the rapid accumulation of navigation error when low-cost MEMS sensors operate without external corrections. This result highlights the limitations of standalone low-cost MEMS sensors in GNSS-denied conditions. As a best-case reference scenario, the yaw-rate channel was replaced with measurements from the high-end reference gyroscope. This substitution reduced the horizontal RMS error to 9.271 m, confirming that heading accuracy is the dominant factor influencing drift in the inertial navigation solution.

**Figure 15:**
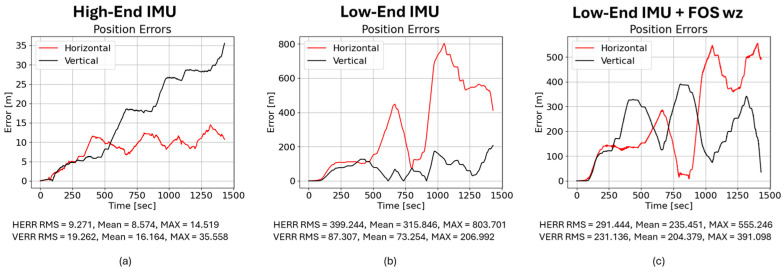
(**a**) High-end IMU yielded horizontal RMS position error = 9.271 m; VERR RMS = 19.262 m. (**b**) Low-end IMU yielded horizontal RMS position error = 399.244 m; VERR RMS = 87.307 m. (**c**) Low-end IMU with FOS-predicted $\omega_{z}$ yielded horizontal RMS position error = 291.444 m; VERR RMS = 231.136 m.

Applying the deterministic FOS correction to the low-end IMU reduced the horizontal RMS error to 291.444 m, corresponding to a 27% improvement relative to the baseline. This result indicates that correcting the dominant gyroscope bias and scale factor errors significantly mitigates heading drift in the inertial navigation solution. Even though the vertical RMS increased (231.136 m), the result highlights that removing the dominant heading bias substantially improves position. Typically, this increase in vertical RMS does not affect navigation for vehicles using roadways, whereas reducing horizontal RMS is important in proper lane adherence. Figure 15 summarizes these three cases.

**Figure 16:**
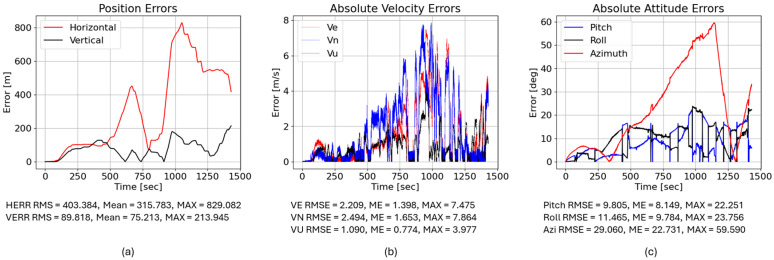
(**a**) Low-end IMU with LSTM-predicted $\omega_{z}$ yielded horizontal RMS position error = 403.384 m; VERR RMS = 89.818 m. (**b**) Absolute velocity errors (east, north, up); LSTM yields VE RMSE = 2.209 m/s, VN RMSE = 2.494 m/s, and VU RMSE = 1.090 m/s. (**c**) Absolute attitude errors; LSTM gives pitch RMSE = 9.805°, roll RMSE = 11.465°, and azimuth RMSE = 29.060°.

We then examined a purely LSTM-based correction (trained on 40,000 samples). Despite its low prediction MSE, it slightly degraded navigation performance: the horizontal RMS error increased to 403.384 m, and the vertical RMS error increased to 89.818 m. This counterintuitive result highlights an important principle in inertial navigation: minimizing signal-level prediction error does not necessarily translate into improved navigation accuracy because small temporal biases or high-frequency fluctuations can accumulate during inertial integration. These results highlight that minimizing signal-level prediction error alone does not guarantee improved inertial navigation performance because the temporal characteristics of residual errors strongly influence drift accumulation during inertial integration. The LSTM output likely introduced small high-frequency fluctuations or phase shifts that were inconsequential in the signal domain but accumulated during INS integration, leading to larger drift. Figure 16 illustrates the LSTM-corrected position, velocity, and attitude errors; note that pitch and roll errors remain moderate, but the azimuth (heading) error still climbs to about 59°, as shown in Figure 16.

**Figure 17:**
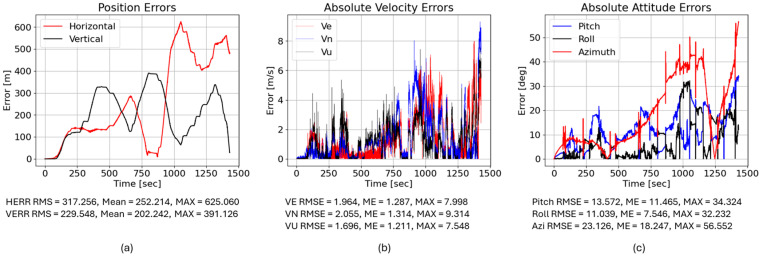
(**a**) Low-end IMU with hybrid-predicted $\omega_{z}$ yielded horizontal RMS position error = 317.256 m; VERR RMS = 229.548 m. (**b**) Absolute velocity errors; hybrid yields VE RMSE = 1.964 m/s, VN RMSE = 2.055 m/s, and VU RMSE = 1.696 m/s. (**c**) Absolute attitude errors; hybrid gives pitch RMSE = 13.572°, roll RMSE = 11.039°, and azimuth RMSE = 23.126°.

Finally, we evaluated the hybrid FOS–LSTM model. Here, the yaw rate $\omega_{z}$ is estimated by a network trained on 6000 real samples plus 34,000 FOS-simulated samples. The hybrid reduced the horizontal RMS error to 317.256 m, which is better than the raw MEMS and LSTM-only corrections but still worse than the FOS-only solution. The vertical RMS error increased to 229.548 m. Interestingly, the hybrid model improved some velocity and attitude metrics relative to the LSTM: the east and north velocity RMSEs dropped from 2.209 and 2.494 m/s to 1.964 and 2.055 m/s, and the azimuth error decreased from 29.060° to 23.126°. This suggests that the hybrid training suppresses high-frequency noise but introduces low-frequency biases that integrate over time. Figure 17 shows the corresponding error traces.

Overall, these results demonstrate that signal-level accuracy alone is insufficient when evaluating inertial sensor corrections. The FOS method provides a smooth, bias-corrected signal that significantly reduces horizontal drift. In contrast, the LSTM and hybrid models achieve low MSE but can introduce temporal artifacts that degrade long-term dead reckoning. For future work, integrating gyroscope corrections with compensations for accelerometer biases and optimizing models for integrated error metrics (rather than MSE) will be essential to fully leverage data-driven approaches in GNSS-denied navigation.

## 6. Discussion

A comparative evaluation of a classical system identification technique (FOS) and a deep learning approach (LSTM) is presented for calibrating low-cost IMU signals. We also proposed a hybrid scheme to combine their strengths. The results reinforce that each approach has distinct advantages and limitations.

FOS excelled at quickly capturing the dominant deterministic error (scale factor bias between the IMUs) with minimal data. Its analytical model was interpretable and remained valid for a new trajectory (Dataset 2) recorded shortly after, indicating stability in that error term. However, FOS is inherently limited by the basis functions chosen; once it accounted for the primary linear bias, any residual complex error (e.g., slight rate-dependent bias changes or higher-order noise patterns) was not modeled, leaving a small gap in accuracy.

Deep LSTM, given enough data, was able to model the IMU’s behavior extremely accurately on the training trajectory. But the LSTM struggled to generalize as well to a new trajectory when trained only on one route, suggesting it overfitted some idiosyncrasies of the training data. The LSTM’s black box nature also means it is not obvious what error it learned to correct. Importantly, the results confirm that minimizing signal-level MSE alone is insufficient for inertial navigation performance, as the temporal characteristics of residual errors critically influence error accumulation.

The hybrid approach was motivated by expecting the best of both worlds: FOS would give the LSTM a leg up by generating a clean dataset reflecting the main bias correction, and the LSTM would fill in the rest. In practice, the hybrid did achieve excellent sensor prediction accuracy with very little real data—matching what an LSTM needed 40,000 points to do. So, in that sense, the data efficiency goal was met. Moreover, the hybrid’s output had lower short-term noise than the LSTM-only model (we saw slightly better velocity errors), indicating that the FOS pre-correction helped the LSTM focus on more subtle patterns. Unfortunately, when it came to position integration, the hybrid did not yet solve the issue of error accumulation—it even slightly worsened it here. We suspect this is because the hybrid’s LSTM learned whatever small discrepancy FOS left, and if that included a tiny bias of opposite sign, it could integrate badly.

This does not mean the concept of the hybrid is flawed—rather, it highlights that simply optimizing for signal fidelity is not enough. One way to improve the hybrid could be to incorporate a cost function that penalizes long-term drift or biases in the LSTM training, effectively teaching it to avoid certain error modes. Another approach could be to perform a joint optimization where the INS performance metric (position drift) is fed back to refine the model.

It is also worth noting that our test scenario (two short trajectories recorded back to back) is a somewhat forgiving case for generalization—environmental changes were minimal. Under more disparate conditions, pure data-driven models might fare even worse without retraining, whereas FOS (if the deterministic error form remains similar) might still hold up until recalibration can be performed. The experiments evaluate cross-trajectory transfer by training on Dataset 1 and testing on Dataset 2, which provides an out-of-sample assessment under different vehicle dynamics. Broader deployment scenarios, such as transferring the learned calibration across different low-cost IMU hardware platforms or across varying environmental conditions (e.g., temperature variation or vibration environments), represent important extensions of this framework. Because MEMS bias and scale factor characteristics can vary with operating conditions, future work will investigate cross-device validation and incorporate environmental variables, such as temperature and vibration, when available, to further evaluate the robustness of the proposed hybrid modeling approach.

The hybrid model did not outperform FOS on the new trajectory, which raises the question: in what situations would the hybrid be most useful? Based on our findings, the hybrid offers an advantage when the amount of real calibration data is very limited, and purely training an LSTM on that would underperform. In our case, with 6 k real points, an LSTM was hopeless, but the hybrid produced a viable model. The deterministic error structure is strong (so FOS can capture a big portion), but there are remaining complex patterns that need more capacity to model. We care more about short-term accuracy (e.g., velocity or incremental pose estimates) than about absolute drift over very long periods. The hybrid clearly improved the short-term error characteristics (sub-percent instantaneous error).

If one’s goal is to minimize long-term drift in a standalone INS, then any approach (hybrid or otherwise) that does not explicitly constrain that drift might fail, as we saw. In practice, one could incorporate domain knowledge into the hybrid—for example, recognizing that the FOS model left a slight bias and compensating for it, or periodically resetting drift if external updates become available.

While our experiments focused on the gyroscopic error (and a brief test on an accelerometer axis, which we will discuss shortly), the concept of combining an analytic model with a learning model is broadly applicable. Any sensor that has a partly predictable error (through physics or calibration) and a partly unpredictable error could benefit. For instance, magnetometer calibration might use a hard-iron bias correction via a model, followed by an LSTM for soft-iron distortions or context-specific noise. The key is identifying a reliable parametric portion and a residual portion best left to a flexible model.

Applying the same FOS–LSTM pipeline to the forward accelerometer $f_{x}$ resulted in higher residual errors (≈20–25% MSE) despite data-efficient training, indicating greater modeling complexity, whereas the gyroscope $\omega_{z}$ achieved sub-0.3% error, demonstrating the hybrid approach’s stronger effectiveness for gyro calibration. The accelerometer results suggest that the hybrid approach is most effective when deterministic error structure dominates, as observed in the gyroscope channel, where bias and scale factor errors are the primary contributors.

## 7. Conclusions

In conclusion, this work advances sensor fusion and calibration for GNSS-denied navigation by demonstrating that integrating system identification with deep learning enables high-performance sensor modeling under limited data conditions. We encourage further exploration of such hybrid methods as the fields of machine learning and sensor systems continue to intersect. This paper presented a data-efficient hybrid calibration framework that combines Fast Orthogonal Search (FOS) with an LSTM network to improve low-cost IMU signal accuracy using a high-grade IMU as reference during a limited labeled interval. The experiments show that FOS can identify dominant deterministic error structure in the yaw rate channel using a small, labeled segment and can produce an interpretable calibration mapping with strong signal-level accuracy. In comparison, LSTM requires substantially more labeled samples to achieve comparable performance, reflecting the data demands of deep sequence learning in this setting.

The proposed hybrid strategy uses FOS-generated pseudo-labels to expand the effective training set for the LSTM stage, enabling the recurrent model to reach high accuracy even when only a short interval of true reference measurements is available. This establishes a practical pathway for deploying deep sequence models in data-constrained calibration workflows: a lightweight identification model provides the initial leverage, and the recurrent model refines residual temporal behavior once sufficient training length is available.

For clarity, the following are the main contributions of this work: (1) a two-stage FOS–LSTM calibration framework for mapping low-cost IMU measurements to high-grade reference outputs; (2) a data-efficient bootstrapping strategy that uses the FOS model as a pseudo-label generator for LSTM training when only a short labeled interval is available; (3) validation on real vehicular datasets using a low-cost MEMS IMU and a tactical-grade reference IMU; (4) an in-system dead reckoning evaluation highlighting that pointwise signal accuracy does not necessarily translate into reduced navigation drift; and (5) interpretability of dominant error structure through analysis of the sparse FOS model terms.

Beyond signal-level accuracy, the navigation experiment highlights that calibration quality should ultimately be assessed in-system; residual error characteristics that are negligible in pointwise metrics can still influence integrated navigation drift. Accordingly, the main outcome is twofold: (i) the hybrid FOS–LSTM framework provides a feasible and effective mechanism for labeled-data expansion in IMU calibration, and (ii) the results motivate navigation-aware learning objectives and constraints to ensure that signal improvements translate reliably into dead reckoning performance. Future work will extend validation across broader temperature/vibration conditions and across different low-cost IMU hardware and will investigate training losses and regularization strategies that explicitly target navigation-relevant error dynamics.

## Figures and Tables

**Table 1:** Sensors used for data collection and reference measurements in the experiments.

Sensor	Specifications
Reference Navigation System (NovAtel PwrPak7-E1)	High-precision GNSS/INS system equipped with a KVH-1750 tactical-grade fiber-optic gyroscope IMU. Provides reference inertial and navigation measurements. Data post-processed using NovAtel Inertial Explorer software (version 8.70, NovAtel Inc., Calgary, AB, Canada). Output synchronized to a 50 Hz timeline.
MEMS IMU (ZED-2i)	Accelerometers: Range ± 8 g, resolution 0.244 mg, noise density 3.2 mg. Gyroscopes: Range ± 1000°/s, resolution 0.03°/s, noise density 0.16°/s. IMU signals were downsampled to 50 Hz during preprocessing to reduce noise and align with the reference data stream.
OBD-II Module (Vehicle Odometer)	Provides forward vehicle speed measurements from the vehicle’s odometer interface. Speed measurements sampled at 3 Hz and used to support vehicle motion analysis during the experiments.

**Table 2:** FOS model results for $\omega_{z}$ (gyro heading rate) at different training data lengths. Percent MSE (%MSE) is evaluated on novel data from Dataset 1 (not used in training). The FOS model equation illustrates the selected terms (with *x*[*n*] being low-end $\omega_{z}$).

z	Training Length (Samples)	%MSE on Test (Dataset 1)	Identified Nonlinear Model
FOS Model 1	5000	76.9966%	*y*[*n*] = 0.0001 + 0.206 * *x*[*n* − 0] −22.7224 * *x*[*n* − 0] * *x*[*n* − 0]
FOS Model 2	6000	0.01458%	*y*[*n*] = 0.0003 + 0.9997 * *x*[*n* − 0]
FOS Model 3	7000	0.007203%	*y*[*n*] = 0.0003 + 0.9991 * *x*[*n* − 0] −0.0008 * *x*[*n* − 0] * *x*[*n* − 0]

**Table 3:** Performance of LSTM-only models versus the hybrid model for predicting high-end $\omega_{z}$. The LSTM models are trained on varying amounts of real high-end data.

Model	Training Length	%MSE (Test)	$R^{2}$ (Test)
LSTM Model 1	5000	83.554%	0.1644
LSTM Model 2	6000	48.72%	0.5128
LSTM Model 3	7000	3.223%	0.9678
LSTM Model 4	10,000	0.3399%	0.9966
LSTM Model 5	40,000	0.0174%	0.9985
Hybrid Model	40,000	0.0139%	0.9999

## Data Availability

The data used in this study are not publicly available.
